# Microtubule-Based Mechanisms of Pronuclear Positioning

**DOI:** 10.3390/cells9020505

**Published:** 2020-02-23

**Authors:** Johnathan L. Meaders, David R. Burgess

**Affiliations:** Department of Biology, Boston College, Chestnut Hill, MA 02467, USA; johnathan.meaders@bc.edu

**Keywords:** dynein, pronucleus, microtubule, MTOC, microtubule aster, zygote, oocyte

## Abstract

The zygote is defined as a diploid cell resulting from the fusion of two haploid gametes. Union of haploid male and female pronuclei in many animals occurs through rearrangements of the microtubule cytoskeleton into a radial array of microtubules known as the sperm aster. The sperm aster nucleates from paternally-derived centrioles attached to the male pronucleus after fertilization. Nematode, echinoderm, and amphibian eggs have proven as invaluable models to investigate the biophysical principles for how the sperm aster unites male and female pronuclei with precise spatial and temporal regulation. In this review, we compare these model organisms, discussing the dynamics of sperm aster formation and the different force generating mechanism for sperm aster and pronuclear migration. Finally, we provide new mechanistic insights for how sperm aster growth may influence sperm aster positioning.

## 1. Introduction

The mature oocyte is the starting point of what eventually becomes a fully developed organism composed of multiple organ systems, multicellular tissues, and a multitude of differentiated and undifferentiated cell types in most animals. The first stage of this transformation begins with one of the most complex transitions in cellular and developmental biology—remodeling the oocyte into a totipotent zygote. Even more noteworthy is the fact that the oocyte contains almost everything required, from mRNA transcripts to molecular signaling proteins and machinery, to guide the oocyte-to-zygote transition [1].

One exception to this maternally dominated “rule” is the paternal contribution of the centrosome during fertilization, which enters the egg with the sperm pronucleus in non-parthenogenetic animals [2]. This sperm-derived microtubule organizing center (MTOC) is essential to restore the diploid condition upon union of the male and female pronuclei, which is the defining feature of the zygote [3]. The transitional period from mature oocyte to zygote is characterized by massive reorganization of the microtubule (MT) cytoskeleton. These MT reorganizations can be subdivided into two general categories: cortical and cytoplasmic. Remodeling the cortical cytoskeleton is centrosome independent and has no known role for union of maternal and paternal pronuclei. Instead, cortical rearrangements are localized with developmental cues important for development [4]. Conversely, cytoplasmic reorganization of the MT cytoskeleton is centrosome-dependent and results in formation of the radial array of MTs, known as the sperm aster. The sperm aster nucleates from the sperm-derived centrosome, which is attached directly to the male pronucleus. Through a process that is still not fully understood, the sperm aster in many animals moves the male pronucleus to the center of the newly fertilized egg where a union of male and female pronuclei occurs prior to mitotic spindle formation. Because the location of pronuclei establishes where the mitotic spindle forms, precise and accurate positioning of the sperm aster and pronuclei within the zygote is critical to determination of the first division axis [5,6].

Precisely how the MT sperm aster generates and responds to forces to move to the cell center can be described by three different mechanisms. The first is through a cortical pulling model in which dynein anchored to the cell cortex attaches to the astral MT plus-ends at the cortex opposite of the side of sperm entry (or front MTs) and generates centering pulling forces through retrograde motility. These MTs also capture the female pronucleus by pulling it to the centrosome of the sperm aster, which results in centration of both male and female pronuclei by the end of sperm aster migration. The second mechanism is through pulling forces generated by retrograde flow of cytoplasmic cargo-bound dynein along astral MTs on all sides of the sperm aster. In this model, termed the MT length dependent cytoplasmic pulling model, a MT length asymmetry within the aster results in more dynein dependent force generation on longer MTs relative to shorter MTs [7]. Accordingly, if MTs at the front of the aster are longer than the rear, then greater pulling forces will be generated in the front relative to the rear and move the sperm aster toward the cell center. The female pronucleus is captured and transported toward the aster center, presumably in a dynein dependent fashion [8]. In this sense, the female pronucleus is also considered as dynein-bound cargo, which also contributes to force generation during centration. The third mechanism is a pushing model in which migration of the sperm aster is dependent on polymerization of rear MTs against the cell cortex on the side of sperm entry. In contrast to the pulling mechanisms, the sperm aster expands to the cell center at a rate that is approximately equal to MT polymerization rates of rear cortical facing MTs and independent of growth rates of front cytoplasmic facing MTs. While cortical and cytoplasmic pulling models for sperm aster positioning have been extensively studied, a pushing model has not yet been observed.

In this review, we will evaluate historical and recent studies, with a focus on reorganization of the MT cytoskeleton into the sperm aster during the oocyte-to-zygote transition, which is pivotal for centration and union of male and female pronuclei. We will compare the primary model organisms in which the sperm aster has been studied in detail, including *Caenorhabditis elegans (C. elegans)*, *Xenopus laevis*, and echinoderms. In this discussion, we will also include the current state of our knowledge of the different force generating mechanism for sperm aster and pronuclear migration and describe how these mechanisms relate to the dynamics of sperm aster formation, including aster geometry, MT growth rates, and proximity to cellular boundaries across model organisms. Finally, we will include an assessment of the current gaps in our knowledge of the topic and outline hypotheses for future studies.

## 2. Sperm Aster Growth and Centration in *C. Elegans*

**Figure 1:**
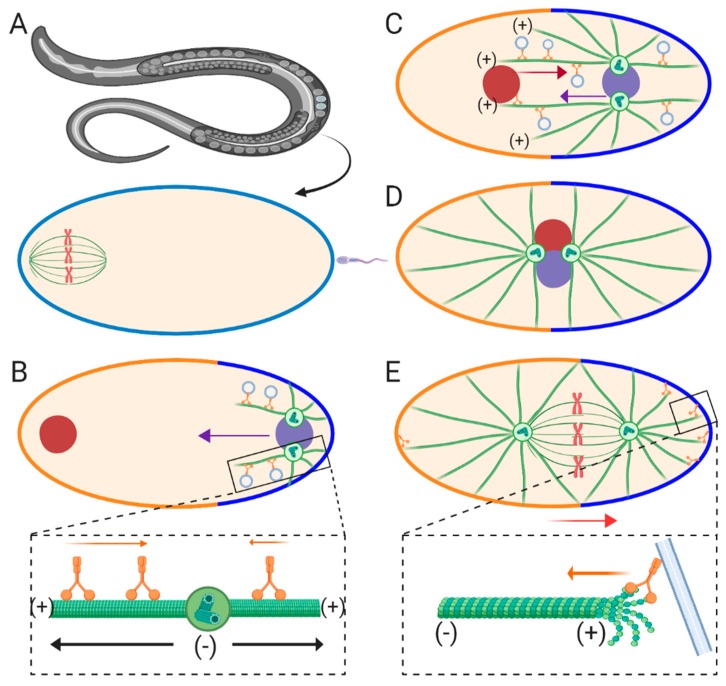
Fertilization and pronuclear migration in *C. elegans*. (**A**) The *C. elegans* oocyte is arrested in metaphase of meiosis I just prior to fertilization. The meiotic spindle is located on the future anterior end of the oocyte, while the sperm/male pronucleus enters on the future posterior end. (**B**) Early centration phase. Fertilization prompts the completion of meiosis and formation of the female pronucleus (red circle). After sperm entry and maturation of the paternally derived centrioles, two sperm asters form oriented on opposite sides of the male pronucleus (purple circle), perpendicular to the anterior-posterior axis. These asters help define the posterior half (bright blue plasma membrane). The asters migrate toward the egg center due to cytoplasmic dynein-dependent pulling forces that scale with MT length (inset). Force (black arrows) is generated in the opposite direction of movement (orange arrows). Therefore, more force is generated on the longer front MTs relative to the short rear/cortical facing MTs. (**C**) Late centration phase. The aster pairs expand during the centration phase, enlarging the posterior half relative to the anterior half of the egg (blue and orange membrane, respectively). The female pronucleus is captured by long front astral MTs and is transported to the male pronucleus by dynein. (**D**) Maintenance phase. The combined male and female pronucleus (pronuclear complex or PNC) finish migrating to the egg center and rotate. This rotation orients centrosomes parallel to the anterior-posterior axis. (**E**) Posteriorization phase. Nuclear envelope breakdown occurs, combining maternal and paternal chromosomes as the first mitotic apparatus forms in the zygote. The apparatus is pulled toward the posterior side by more dynein activity at the posterior half relative to the anterior (inset). MT catastrophe is also considered as a potential mechanism to generate forces (inset).

Due to its powerful genetic tool kit and optically tractable eggs, *C. elegans* is one of the most thoroughly studied models for the assembly and migration of sperm asters. *C. elegans* eggs represent a ~50 μm oval-shaped cell type, consisting of the future anterior end containing the maternal meiotic spindle and the future posterior end where fertilization and entry of the male pronucleus occurs (Figure 1A) [9]. The unfertilized egg is arrested in meiosis I, which resumes upon fertilization resulting in formation of the female pronucleus upon meiotic completion [10]. During the time period between fertilization and formation of the female pronucleus, maturation of the paternal centriole is suppressed and held in place at the posterior cortex by F-actin and kinesin-1 in order to prevent premature capture of the meiotic spindle by the sperm aster [11,12]. After fertilization and completion of meiosis II, centrosome maturation occurs due to recruitment of γ-tubulin and other maternally supplied factors, resulting in dynein-dependent separation of the centrosomes and sperm aster formation (Figure 1B) [13,14,15,16]. The morphology of the sperm aster in this model was first observed by immunofluorescence revealing two MTOCs attached to the male pronucleus at the future posterior end of the cell [17]. These centrosomes migrate to opposite sides of the male pronucleus, orienting their bipolar axis perpendicular to the anterior-posterior axis at the onset of the first mitotic prophase. This centrosome pair then nucleates MTs which contact the nearby cortex behind the male pronucleus [17], which was later found to deliver determinants to establish the posterior-anterior axis (Figure 1B) [18]. As these aster pairs begin to grow, an early aster asymmetry becomes apparent. Front MTs oriented toward the anterior side of the egg are longer than rear MTs growing toward the posterior side, interacting with the cortex [17]. These longer front MTs are responsible for capturing and transporting the female pronucleus toward the male pronucleus in a dynein-dependent manner (Figure 1C) [19]. Around this time point, the sperm aster pair, along with the female pronucleus (termed the pronuclear complex or PNC), migrates toward the cell center. During this phase, known as the centration phase, the sperm asters orient perpendicular to the anterior-posterior axis, located between the male and female pronuclei. As the PNC approaches the cell center rotation of the asters occurs, orienting them parallel with the anterior-posterior axis (Figure 1D). Finally, the PNC is displaced posteriorly, as the first mitotic spindle begins to form, resulting in the diploid zygote (Figure 1E).

The force generating mechanisms governing centration and posterior migrations in *C. elegans* have been systematically investigated in a series of genetic loss of function studies. First, it was established that dynein and MTs are required for faithful aster centration in this system [14,15,16], which suggests that a pulling mechanism along MTs is the predominant force driver. Which pool of dynein, cortical, cytoplasmic, or a combination of both, contributes to aster centration has been a topic of numerous studies within the field. RNAi-mediated inhibition of cortical factors required for dynein recruitment results in faster migration of sperm asters during centering [17,18], while posterior displacement after rotation of the PNC is abrogated [17,19,20,21,22]. These studies indicate that cytoplasmic dynein is the primary candidate for generating centering pulling forces on the sperm asters during centration, which are counteracted by cortical pulling forces (Figure 1B inset). These cortical pulling forces then take over to displace the forming mitotic spindle during posterior movements (Figure 1E inset) [23,24,25,26]. Furthermore, because total dynein inhibition abrogates aster centration, it’s believed that MT polymerization against the cortex does not significantly contribute to sperm aster migration [15]. Conversely, a more recent study using magnetic tweezers to pull the aster pair anteriorly or posteriorly after aster migration is complete implicates spring-like forces which maintain the position of aster pairs, which is consistent with MT-based pushing mechanisms [27]. Finally, while kinesin-1 is required to prevent premature centrosome maturation and pronuclear migration [12], it is still unknown if kinesins-1 and/or other kinesins are essential for pronuclear migration during the centration phase, which would implicate potential and substantial motor-driven pushing forces.

In a cytoplasmic dynein-dependent pulling model, retrograde movement of dynein/cargo is expected to generate pulling forces on all sides of the aster [7,28]. How does pulling on all sides of the sperm aster translate into directionally applied forces and migration rates? The asymmetric geometry of the sperm aster during the centration phase reveals longer MTs in front of the centrosome pair growing deep into the cytoplasm compared to the rear MTs limited by the posterior cortex. If cytoplasmic dynein-dependent force scales with MT length, then we can assume that more force will be generated along the front astral MTs relative to the rear, driving aster migration in the direction of the longest MTs [7,25] (Figure 1B inset). This cytoplasmic MT length-dependent pulling hypothesis was first modeled in silico using *C. elegans* [29]. Computer simulations predict that in the MT length-dependent pulling model, migration rates of the sperm aster pair will fit a sigmoidal curve when plotted as migration distance vs. time. Conversely, a pushing model should display a convex curve in which rates positively scale with the number of MTs polymerizing against the rear cortex [29]. While tracking PNC migration during the centration phase, rates match a sigmoidal curve suggesting that the asters are being pulled by cytoplasmic pulling forces, which positively scale with MT-length. These sigmoidal migration dynamics were more recently confirmed by an independent study, which also showed an increase in migration rates upon removal of cortical antagonistic factors, providing compelling evidence for a MT-length dependent cytoplasmic pulling model during the centration phase in *C. elegans* [22]. However, the MT growth rate parameters used to simulate migration curves in a pushing model assume non-variable MT growth rates [29]. While MT growth rates have not been measured with precise temporal resolution during the centration phase, average MT growth rates during early pronuclear migration are highly variable [30]. An alternative, untested hypothesis is that MT growth rates start off slow as the sperm asters are forming, then increase during the bulk of the migration phase, and slow down as the sperm aster approaches the egg center, which would also result in a sigmoidal migration curve for a pushing model. Future work measuring MT growth rates with high temporal resolution throughout the aster centration phase in *C. elegans* will help test this hypothesis. Finally, what are the exact membrane bound cytoplasmic cargoes that anchor dynein in order to generate MT-length dependent pulling forces? Evidence for endocytic transport is implicated in generating cytoplasmic pulling forces [31]. By inhibiting different Rab-coated endocytic transport, it was shown that the PNC moves at a slower rate during the centration phase. Furthermore, centration rates of the sperm aster pair are increased when retrograde transport of the largest cargo, the female pronucleus, is inhibited in a background lacking cortical antagonistic factors [22]. Another cytoplasmic dynein/cargo interaction that could result in pulling forces on the sperm aster is dynein-mediated transport of the endoplasmic reticulum (ER). By interacting with the ER through membrane contact sites, endomembrane compartments, such as lysosomes bound to dynein, may transport the endomembrane and its associated ER retrograde toward the MTOC [32]. These endomembrane/ER interactions increase the size and drag of the cytoplasmic anchor for dynein, which in turn should increase the amount of effective pulling forces each dynein motor may generate on the sperm aster. Indeed, ER has been shown to undergo massive retrograde migration and accumulation around the centrosomes of the centering sperm aster [33], making it a strong candidate for generating cytoplasmic pulling forces. Future work focusing on other cargoes will be required to elucidate the identity of new cytoplasmic cargo and the specific contributions of different cargoes required for generating cytoplasmic pulling forces.

Generation of pulling forces due to cortical factors during the posterior-directed movements of the asters after centration are relatively straightforward upon initial observation. That is, cortically bound dynein can anchor astral MTs and generating pulling forces through retrograde motility (Figure 1E inset). However, how dynein moves the centered asters specifically toward the posterior side of the egg is more complex. This problem is solved by an asymmetric distribution of dynein at the cortex in which dynein is more concentrated along the posterior half than the anterior [34]. Therefore, more dynein-dependent pulling forces are generated on the posterior side of the egg than the anterior, resulting in a shift of the aster pair posteriorly (Figure 1E). A second potential mechanism used to generate pulling forces is depolymerization of cortically anchored MT plus-ends (Figure 1E inset) [26,35]. Experiments using taxol to study the role of MT dynamics in these posterior movements suggest that regulated MT depolymerization may be responsible for generating the required pulling forces [26]. Other work shows a strong correlation between MT catastrophe and aster movement [35]. MT depolymerization-dependent pulling was directly shown more recently in vitro. Dynein was artificially anchored to a barrier, where it was directly shown to attach and negatively regulate the lengths of MT lengths [36]. However, a potential role for dynein-dependent catastrophe-mediated pulling during aster positioning has not yet been directly characterized in vivo using a developmental model.

## 3. Sperm Aster Growth and Centration in Echinoderms

**Figure 2:**
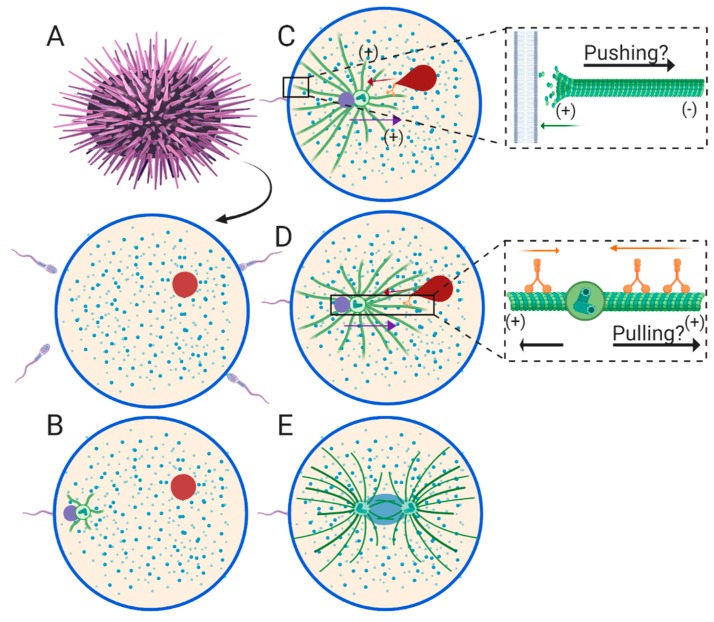
Fertilization and pronuclear migration in the sea urchin (echinoderm). (**A**) The sea urchin oocyte has already completed meiosis resulting in formation of the female pronucleus (red circle), which is located randomly within the oocyte cytoplasm. Fertilization may also occur anywhere around the oocyte. (**B**) Almost immediately after fertilization, the paternally-derived centrosome is attached to the male pronucleus (purple circle) and begins forming the interphase sperm aster near the cortex. During this early time-point the sperm aster does not begin to migrate until astral MTs reach the rear cortex. (**C** and **D**) As the sperm aster grows, it enters the centration phase where it reaches a constant maximum speed. This velocity is either set by growth rates of rear cortical facing MTs pushing against the cortex as in (**C**), cytoplasmic dynein-dependent pulling forces that scale with MT lengths as in (**D**), or a combination of the two. The female pronucleus is captured by astral MTs and is presumably transported towards the aster center/male pronucleus by dynein. Transport causes the female pronucleus to form a “tear drop” shape (**E**) The sperm aster slows down as it approaches the egg center, prophase centrosomes separation occurs, and pronuclei fuse forming the zygote nucleus (blue oval).

In contrast to *C elegans*, the echinoderm egg is a perfectly spherical, ~80~200 μm diameter, non-polarized oocyte. Additionally, the oocyte of some echinoderms, such as sea urchins, have already completed meiosis before fertilization occurs (Figure 2A), which results in stark differences compared to *C. elegans*. In the sea urchin, the female pronucleus has already formed in the mature oocyte, and can be located anywhere in the cytoplasm [37]. Similarly, fertilization occurs at spatially indiscriminate locations around the oocyte plasma membrane (Figure 2A). Therefore, the male and female pronuclei are positioned at random locations relative to each other just after fertilization, rather than at opposite poles as in *C. elegans*. Because of this initial location, engagement between the male and female pronucleus also occurs at seemingly random time points after fertilization, sometimes resulting in fusion of the male and female pronucleus before centration has even been completed. The engagement between the sperm aster and the female pronucleus and subsequent retrograde transport is presumably dynein-dependent in echinoderms (Figure 1C,D). However, direct testing of this hypothesis has yet to be performed. Another difference when compared to *C. elegans* is that there is no requirement for sperm aster formation and migration to be delayed while the maternal chromosomes complete meiosis in sea urchin eggs. Accordingly, centrosome maturation, sperm aster growth, and migration begin almost immediately after the male pronucleus enters the egg cytoplasm [38].

Initial immunofluorescence observation of the echinoderm sperm aster revealed an interphase sperm monaster, which appears to expand as it approaches the cell center [39,40,41]. These early studies describe three phases of sperm aster migration distinguished by different migration rates, throughout the centration process. The first phase is just after fertilization (Figure 2B), when the asters can be described as “small stars” [41] and move at a rate of ~3.5 μm/min [42]. Another independent study indicates that the aster during this phase has a symmetrical geometry, as it is beginning to grow [38]. The second phase consists of the bulk of aster expansion and the majority of the movement toward the egg center at rates of ~4.9 μm/min. Bright field microscopy of aster geometry during this phase describes an asymmetric aster geometry in which rear/cortical MTs grow at a faster rate than front MTs leading into the cytoplasm, which is consistent with a pushing model (Figure 2C) [38]. A later independent study using DIC microscopy indicates that the male pronucleus does not begin moving until the expansion of rear MTs reaches and grows against the rear cortex, leading to the conclusion that the aster is pushed to the cell center [43]. During the third phase, the aster slows down to ~2.6 μm/min as it nears the center, and centrosome separation around the newly formed zygote nucleus results in two large asters that completely fill the cytoplasm (Figure 2E). These three phases of aster migration were recently reconfirmed in an independent study using updated methods for tracking sperm aster migration [44].

One caveat of echinoderms as a model system is they lack genetic tools to study aster formation and pronuclear migration. However, because they are very malleable, clear, and not yet polarized, echinoderm eggs represent a powerful live-cell system to study the biophysical principles of how aster geometry translates to migration rates and directional forces. Previous work describes a prominent network of astral MTs extending to the cortex, which were originally predicted to push the sperm aster to the cell center (Figure 2C) [40]. Additionally, MTs do not reach the far opposite cortex until the third phase of aster migration, when centrosome separation occurs and migration rates come to a halt (Figure 2E), indicating that cortical pulling mechanisms are not a contributing factor. However, subsequent work using the MT inhibitor, colcemid, weakened this pushing hypothesis in sand dollar eggs [7]. In a hallmark study, eggs were treated with colcemid and then fertilized. Following fertilization, colcemid was deactivated with UV irradiation in a 50–60 μm diameter region of the egg containing the male pronucleus. When the male pronucleus is at the periphery of the irradiated region, it migrates toward the geometric center of the region where it comes to a halt. In other words, male pronuclear migration occurs in the direction of the longest astral MTs until it reaches the center of the irradiated region, where MT lengths are presumably equal on all sides of the aster (Figure 2D inset). These experiments provided the first evidence for a MT length-dependent cytoplasmic pulling mechanism in any model organism [7].

More recently, modern techniques utilizing laser ablation, magnetic tweezers and in silico modeling have revisited the MT-length dependent pulling model, investigating how such a model accounts for aster migration direction and speeds in the sea urchin [44,45,46,47]. Laser ablation of side portions of the sperm aster results in drift of the male pronucleus away from the side of ablation in a MT-dependent manner, indicating that it is being pulled from the opposite side where MTs are theoretically longer [44]. Likewise, by using magnetic tweezers, the aster is pulled perpendicular to the centration path. When the magnets are released, the aster resumes migration toward the cell center, in the direction of the theoretically longest MTs [45]. Together, these series of experiments suggest that aster directionality is maintained by forces on side astral MTs that scale with MT length. Additionally, ablations along front, cytoplasmic facing MTs results in momentary pauses in aster forward migration [44], suggesting pulling forces at the front of the aster. Mathematical and computational modeling of the sperm aster in this same study suggests that aster migration rates are determined by growth rates of the sperm asters, where speed is equal to the length of front astral MTs minus the length of rear astral MTs (Figure 2D inset). Together, this body of literature suggests MT-length dependent pulling forces driven by cytoplasmic dynein are predominant during aster migration and centration in echinoderms. However, while global inhibition of dynein using Ciliobrevin D halts aster migration in the sea urchin, inhibition of dynein during laser ablation, magnetic redirection, and colcemid experiments has not yet been tested [7,44,45]. Therefore, the presumed role of dynein in the observed movements away from the site of ablation, away from the released magnets, and toward the center of UV irradiated colcemid regions, respectively, is currently unknown. Moreover, while side and front astral MTs have been manipulated in these studies, experiments manipulating the MTs growing against the rear cortex at the site of sperm entry have not been conducted. Such manipulation experiments will more directly test if MT pushing may drive aster forward migration [3]. Finally, the MT length dependent pulling model critically depends on a particular aster geometry in which the front/cytoplasmic facing radius must be longer than the rear/cortical facing radius (Figure 2D) [44]. Earlier characterization of aster geometry using bright field and DIC microscopy suggests that the rear/cortical radius of the aster expands faster than the front radius during the migration phase, which is consistent with a pushing model and challenges the MT-length dependent pulling model (Figure 2C) [38]. However, modern approaches to characterize sperm astral MT lengths and dynamics in live cells have not yet been reported. These measurements will prove particularly important for thoroughly investigating these conflicting models.

## 4. Sperm Aster Growth and Centration in *Xenopus*

**Figure 3:**
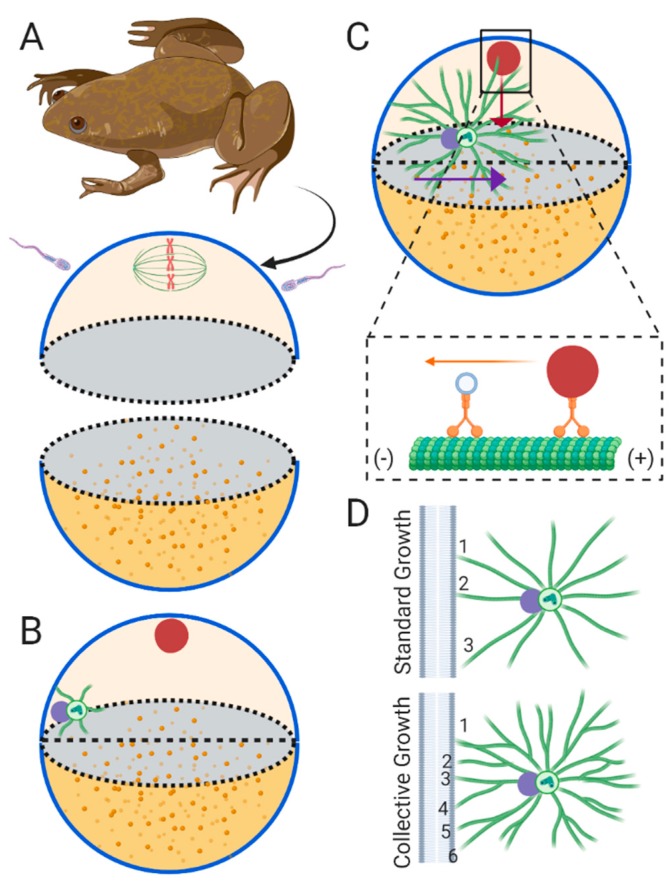
Fertilization and pronuclear migration in *Xenopus.* (**A**) The frog oocyte is arrested in metaphase II of meiosis. The meiotic spindle is located at the pole of the animal half of the egg (top beige hemisphere). The sperm can fertilize the egg along the side of the animal half. The yolky vegetal half is illustrated as the lower dark yellow hemisphere. (**B**) Fertilization resumes the cell cycle, resulting in formation of the female pronucleus (red circle) near the animal pole after meiosis completes. The paternally derived centrosomes begin forming the interphase sperm aster attached to the male pronucleus (purple circle). (**C**) The sperm aster expands and migrates toward the center of the egg, just above the vegetal half. As the astral MTs contact the female pronucleus it is transported retrograde along astral MTs in a dynein dependent manner (inset). Furthermore, cytoplasmic dynein/cargo (inset) likely generates pulling forces through retrograde transport. (**D**) Simplification of sperm aster growth according the standard growth model (top) and the collective growth model (bottom). The standard growth model predicts that asters are formed solely from centrosome-nucleated MTs, while the collective growth model includes MT-dependent MT nucleation, or MT branching. When considering pushing forces due to MT polymerization against the cell cortex, long individual MTs (numbered 1–3) nucleate from the centrosome and bear a high compression load, which can lead to MT buckling and decentering (see text for details). However, this problem is solved by the collective growth model in which the compression load is redistributed to a greater number of short MTs (numbered 1–6) polymerizing against the cortex.

Amphibian eggs represent extremely large cells, sometimes reaching diameters of up to 1 mm. Accordingly, pronuclei must undergo extremely long migration distances compared to *C. elegans* and echinoderms. The earliest accurate studies of pronuclear migration dynamics were performed in the amphibian [48]. Before fertilization, the egg is arrested in metaphase II of meiosis, much like in *C. elegans*, and the meiotic spindle is located at the animal pole (Figure 3A). Fertilization occurs randomly along the animal half of the egg and triggers completion of meiosis, resulting in formation of the female pronucleus. Meanwhile, paternal centrosomes carried by the sperm nucleate the interphase sperm aster (Figure 3B). Immunofluorescence microscopy of the sperm aster reveals massive expansion into the egg cytoplasm within the animal pole, which eventually captures the female pronucleus (Figure 3C) [49]. The sperm aster then carries the male and female pronuclei toward the center of the egg, just above the yolk-dense vegetal half. Here, onset of the first mitosis occurs, and fusion of the maternal and paternal DNA completes, forming the diploid zygote [50].

Due to the opaque properties of the frog egg, modern live-cell investigations of sperm aster growth and migration dynamics are notably limited. However, experiments combining microinjection and fixed-cell immunofluorescence microscopy have shed light on how the sperm aster positions pronuclei at the cell center. As the sperm aster expands, MT lengths are restricted by the cortex proximal to the site of sperm entry [49]. Conversely in front of the aster, MTs are not near long enough to contact the opposite cortex ruling out a cortical pulling model. Therefore, much like in *C. elegans* and sea urchins, the centration mechanisms are likely due to either pushing from MT polymerization against the rear membrane or from pulling in the cytoplasm by dynein bound to its cargo. To test for dynein-dependent pulling, eggs were injected with a dominant negative fragment of the dynactin complex (p150-CC1) after fertilization and processed for immunofluorescence microcopy at varying time-points post-fertilization. Injected eggs displayed reduced sperm aster migration dynamics when compared to controls. Furthermore, aster morphology in injected eggs display centrosomes still near the cortex, with a longer front aster radius reaching into the cytoplasm, and a shorter rear aster radius limited by the rear cortex [49]. Together, these experiments provide strong evidence indicating that dynein in the cytoplasm is required to pull the sperm aster to the egg center (Figure 3C).

While live cell experimentation in amphibian eggs is challenging, the use of *Xenopus* egg extracts provides a powerful model for *in vitro* studies of aster growth dynamics and positioning of male and female pronuclei [51,52]. The requirement of dynein during female pronuclear translocation along MTs were first directly tested in *Xenopus* interphase egg extracts [8]. Magnetic beads were used to bind DNA and form an artificial nucleus lacking a centrosome. These nuclei move along MTs toward purified centrosomes ends at rates comparable to those measured during female pronuclear migration in echinoderms [42], and inhibition of dynein using blocking antibodies or vanadate abrogates these movements. Importantly, the extracts in which purified nuclei and centrosomes were diluted consists of the cytoplasm taken directly from interphase eggs, providing strong support that female pronuclear migration along interphase sperm asters is dynein dependent (Figure 3C). Determining if *Xenopus* female pronuclear migration along the sperm aster is dynein dependent in vivo may prove challenging because dynein also appears to be required for migration of the sperm aster. Additionally, the mechanisms required for precise control of migration and positioning of large interphase sperm aster using *Xenopus* extracts has not yet been tested. By using micro-fabricated chambers matching the sizes and shapes of eggs from different model organisms, these extracts will provide a rich reconstitution system for uncovering the exact contribution of differing mechanisms during sperm aster centration.

More recently, *Xenopus* extracts have prompted a reconsideration for how large MT asters grow in developmental systems. So far we have only considered astral MTs nucleated from the paternally inherited centrosome, also known as the radial elongation model of aster growth (Figure 3D top) [53]. However, work using both interphase and meiotic *Xenopus* egg extracts has led to the discovery that these especially large asters nucleate MTs remote from the centrosome, termed the collective growth model (Figure 3D bottom) [54,55]. In meiotic egg extracts, these centrosome-independent MT nucleation events occur through a process of MT-dependent MT nucleation, or MT branching [54]. The first question that the collective model answers is how can an aster radius span the large cytoplasm of large oocytes after fertilization? In the radial elongation model, this would mean individual centrosome-nucleated MTs, whose lengths are bound by dynamic instability at their plus-ends [56], must grow hundreds of microns in lengths to span the cytoplasm. However, in the collective growth model, parental MTs nucleated at the centrosome nucleate subsequent daughter MTs along their sides, and these daughter MTs may then nucleate new MTs in a branched network spanning large distances (Figure 3D bottom). This branching was also recently predicted to account for the increase in MT density observed in the *Xenopus* sperm aster at distances remote from the centrosome in fixed immunofluorescence images. That is, the number of MTs increases as a function of distance from the centrosome [55,57]. While elaborate in vitro studies are currently focusing on the mechanisms and dynamics of MT branching during aster growth [58,59,60,61,62], future work determining if sperm asters contain branched MTs in vivo will be required.

The collective growth model also has strong implications for the mechanisms required for sperm aster positioning and pronuclear migration. First, one limitation to the pushing model for aster positioning in large asters is the extremely high number of MTs that would be required to push a large sperm aster over large distances through the highly viscous cytoplasm. This number was estimated to be approximately 12,000 MTs midway through centration in *Xenopus* sperm asters [3]. Such a high estimate is partially due to data indicating that as MTs become longer they tend to buckle, resulting in a loss of centering forces (Figure 3D bottom) [63,64,65,66]. Conversely, we expect that the compression load would be redistributed among many shorter branched MTs growing at the cortex in a collective growth model, rather among long individual MTs nucleated from the centrosome in a radial elongation model (Figure 3D). This redistribution of the compression load across a network of branched astral MTs, should reduce the required number of MT polymerization events at the cell cortex to move large sperm aster. Additionally, MT branching should result in more MT polymerization events occurring against the cell cortex when compared to the standard growth model (Figure 3D). Modeling how this force would be redistributed among a branched network and how many polymerization events would be required to generate enough pushing will be required to test this hypothesis. A second implication to consider is retrograde transport of organelles, including the female pronucleus, along a branched network of MTs. In other words, how can transport of cargo ranging from small vesicles to the large female pronucleus occur through a dense network of branched astral MTs? One hypothesis is that dynein and its bound cargo can switch from one MT to another during migration [67]. However, in such a model, whether or not the female pronucleus can maintain the recorded migration rates (~0.24 μm/second) is unknown.

## 5. Conclusions

Union of male and female pronuclei is a defining feature of the oocyte-zygote transition during very early development in non-parthenogenetic animals. In most animals, massive rearrangements of the MT cytoskeleton form the sperm aster, which is essential for migration and positioning of pronuclei during this transition. Decades of research using *C. elegans*, echinoderm, and *Xenopus* eggs suggests a relatively conserved mechanism in which sperm aster positioning is dominated by dynein dependent pulling forces in the cytoplasm that may scale with MT length. Despite the major evolutionary differences between the model organisms presented here, all three have adopted a pulling mechanism, which appears essential for aster and pronuclear positioning. From an evolutionary perspective, this is likely due to the relatively large size of zygotes requiring long migration distances for the sperm aster and pronuclei to reach the cell center. These distances present physical constraints when considering a pushing model (Figure 3D), which may have resulted in convergence of these organisms on a pulling model.

The potential for collective growth during aster formation may solve the physical constraints on long range migration of MT structures such as the sperm aster, making the argument for pushing based-mechanisms far more plausible (Figure 3D). While the idea that dynein function is essential for pronuclear migration has been well established, whether or not dynein is sufficient is still an important unanswered question. That is, are there any roles for MT-based and/or motor based pushing models during aster migration? If the sperm aster lacks potential pushing factors such as rear/cortical MTs or kinesin function, can dynein-dependent pulling still move the sperm aster to the cell center with the pronuclei in tow? If not, pushing mechanisms may be just as important to position pronuclei as dynein-dependent pulling. Future work focusing on the rear cortically oriented MTs and perturbing different kinesins will key to determining whether or not pushing forces during aster positioning may also be essential.

## References

[1] Sha Q.-Q., Zhang J., Fan H.-Y. (2019). A story of birth and death: mRNA translation and clearance at the onset of maternal-to-zygotic transition in mammals. Biol. Reprod..

[2] Schatten G. (1994). The Centrosome and Its Mode of Inheritance: The Reduction of the Centrosome during Gametogenesis and Its Restoration during Fertilization. Dev. Biol..

[3] Reinsch S., Gonczy P. (1998). Mechanisms of nuclear positioning. J. Cell Sci..

[4] Nishikata T., Goto T., Yagi H., Ishii H. (2019). Massive cytoplasmic transport and microtubule organization in fertilized chordate eggs. Dev. Biol..

[5] Pollard T.D., O’Shaughnessy B. (2019). Molecular Mechanism of Cytokinesis. Annu. Rev. Biochem..

[6] Rappaport R. (1961). Experiments concerning the cleavage stimulus in sand dollar eggs. J. Exp. Zool..

[7] Hamaguchi M.S., Hiramoto Y. (1986). Analysis of the Role of Astral Rays in Pronuclear Migration in Sand Dollar Eggs by the Colcemid-UV Method. Dev. Growth Differ..

[8] Reinsch S., Karsenti E. (1997). Movement of nuclei along microtubules in Xenopus egg extracts. Curr. Biol..

[9] Wu Y., Griffin E.E. (2017). Regulation of Cell Polarity by PAR-1/MARK Kinase. Curr. Top. Dev. Biol..

[10] McNally K.L., McNally F.J. (2005). Fertilization initiates the transition from anaphase I to metaphase II during female meiosis in C. elegans. Dev. Biol..

[11] Panzica M.T., Marin H.C., Reymann A.-C., McNally F.J. (2017). F-actin prevents interaction between sperm DNA and the oocyte meiotic spindle in C. elegans. J. Cell Biol..

[12] McNally K.L.P., Fabritius A.S., Ellefson M.L., Flynn J.R., Milan J.A., McNally F.J. (2012). Kinesin-1 Prevents Capture of the Oocyte Meiotic Spindle by the Sperm Aster. Dev. Cell..

[13] Kemp C.A., Kopish K.R., Zipperlen P., Ahringer J., O’Connell K.F. (2004). Centrosome Maturation and Duplication in C. elegans Require the Coiled-Coil Protein SPD-2. Dev. Cell..

[14] Pelletier L., O’Toole E., Schwager A., Hyman A.A., Müller-Reichert T. (2006). Centriole assembly in Caenorhabditis elegans. Nature.

[15] Gönczy P., Pichler S., Kirkham M., Hyman A.A. (1999). Cytoplasmic Dynein Is Required for Distinct Aspects of Mtoc Positioning, Including Centrosome Separation, in the One Cell Stage Caenorhabditis elegans Embryo. J. Cell Biol..

[16] Hamill D.R., Severson A.F., Carter J.C., Bowerman B. (2002). Centrosome Maturation and Mitotic Spindle Assembly in C. elegans Require SPD-5, a Protein with Multiple Coiled-Coil Domains. Dev. Cell.

[17] Albertson D.G. (1984). Formation of the first cleavage spindle in nematode embryos. Dev. Biol..

[18] Lang C.F., Munro E. (2017). The PAR proteins: From molecular circuits to dynamic self-stabilizing cell polarity. Development.

[19] Malone C.J., Misner L., Bot N.L., Tsai M.-C., Campbell J.M., Ahringer J., White J.G. (2003). The C. elegans Hook Protein, ZYG-12, Mediates the Essential Attachment between the Centrosome and Nucleus. Cell.

[20] Zipperlen P., Fraser A.G., Kamath R.S., Martinez-Campos M., Ahringer J. (2001). Roles for 147 embryonic lethal genes on C.elegans chromosome I identified by RNA interference and video microscopy. EMBO J..

[21] Kimura A., Onami S. (2007). Local cortical pulling-force repression switches centrosomal centration and posterior displacement in C. elegans. J. Cell Biol..

[22] De Simone A., Spahr A., Busso C., Gönczy P. (2018). Uncovering the balance of forces driving microtubule aster migration in C. elegans zygotes. Nat. Commun..

[23] Gotta M., Ahringer J. (2001). Distinct roles for Gα and Gβγ in regulating spindle position and orientation in Caenorhabditis elegans embryos. Nat. Cell Biol..

[24] Lorson M.A., Horvitz H.R., van den Heuvel S. (2000). LIN-5 Is a Novel Component of the Spindle Apparatus Required for Chromosome Segregation and Cleavage Plane Specification in Caenorhabditis elegans. J. Cell Biol..

[25] Gotta M., Dong Y., Peterson Y.K., Lanier S.M., Ahringer J. (2003). Asymmetrically Distributed C. elegans Homologs of AGS3/PINS Control Spindle Position in the Early Embryo. Curr. Biol..

[26] Nguyen-Ngoc T., Afshar K., Gönczy P. (2007). Coupling of cortical dynein and Gα proteins mediates spindle positioning in Caenorhabditis elegans. Nat. Cell Biol..

[27] Garzon-Coral C., Fantana H.A., Howard J. (2016). A force-generating machinery maintains the spindle at the cell center during mitosis. Science.

[28] Shinar T., Mana M., Piano F., Shelley M.J. (2011). A model of cytoplasmically driven microtubule-based motion in the single-celled Caenorhabditis elegans embryo. Proc. Natl. Acad. Sci. USA.

[29] Kimura A., Onami S. (2005). Computer Simulations and Image Processing Reveal Length-Dependent Pulling Force as the Primary Mechanism for *C. elegans* Male Pronuclear Migration. Dev. Cell.

[30] Srayko M., Kaya A., Stamford J., Hyman A.A. (2005). Identification and Characterization of Factors Required for Microtubule Growth and Nucleation in the Early C. elegans Embryo. Dev. Cell.

[31] Kimura K., Kimura A. (2011). Intracellular organelles mediate cytoplasmic pulling force for centrosome centration in the Caenorhabditis elegans early embryo. Proc. Natl. Acad. Sci. USA.

[32] Bonifacino J.S., Neefjes J. (2017). Moving and positioning the endolysosomal system. Curr. Opin. Cell Biol..

[33] Terasaki M., a Jaffe L. (1991). Organization of the Sea-Urchin Egg Endoplasmic-Reticulum and Its Reorganization at Fertilization. J. Biophys. Biochem. Cytol..

[34] McNally F.J. (2013). Mechanisms of spindle positioning. J. Cell Biol..

[35] Kozlowski C., Srayko M., Nedelec F. (2007). Cortical Microtubule Contacts Position the Spindle in C. elegans Embryos. Cell.

[36] Laan L., Pavin N., Husson J., Romet-Lemonne G., van Duijn M., López M.P., Vale R.D., Jülicher F., Reck-Peterson S.L., Dogterom M. (2012). Cortical Dynein Controls Microtubule Dynamics to Generate Pulling Forces that Position Microtubule Asters. Cell.

[37] Peng C.J., Wikramanayake A.H. (2013). Differential Regulation of Disheveled in a Novel Vegetal Cortical Domain in Sea Urchin Eggs and Embryos: Implications for the Localized Activation of Canonical Wnt Signaling. PLoS ONE.

[38] Chambers E.L. (1939). The Movement of the Egg Nucleus in Relation to the Sperm Aster in the Echinoderm Egg. J. Exp. Biol..

[39] Bestor T.H., Schatten G. (1981). Anti-tubulin immunofluorescence microscopy of microtubules present during the pronuclear movements of sea urchin fertilization. Dev. Biol..

[40] Hamaguchi Y., Toriyama M., Sakai H., Hiramoto Y. (1985). Distribution of fluorescently labeled tubulin injected into sand dollar eggs from fertilization through cleavage. J. Cell Biol..

[41] Harris P., Osborn M., Weber K. (1980). Distribution of tubulin-containing structures in the egg of the sea urchin Strongylocentrotus purpuratus from fertilization through first cleavage. J. Cell Biol..

[42] Schatten G. (1981). Sperm incorporation, the pronuclear migrations, and their relation to the establishment of the first embryonic axis: Time-lapse video microscopy of the movements during fertilization of the sea urchin Lytechinus variegatus. Dev. Biol..

[43] Hamaguchi M.S., Hiramoto Y. (1980). Fertilization Process in the Heart-Urchin, Clypeaster Japonicus Observed with a Differential Interference Microscope. Dev. Growth Differ..

[44] Tanimoto H., Kimura A., Minc N. (2016). Shape–motion relationships of centering microtubule asters. J. Cell Biol..

[45] Tanimoto H., Sallé J., Dodin L., Minc N. (2018). Physical forces determining the persistency and centring precision of microtubule asters. Nat. Phys..

[46] Minc N., Burgess D., Chang F. (2011). Influence of Cell Geometry on Division-Plane Positioning. Cell.

[47] Sallé J., Xie J., Ershov D., Lacassin M., Dmitrieff S., Minc N. (2018). Asymmetric division through a reduction of microtubule centering forces. J. Cell Biol..

[48] Roux W. (1885). Die Entwickelungsmechanik der Organismen. Wien..

[49] Wühr M., Tan E.S., Parker S.K., Detrich H.W., Mitchison T.J. (2010). A Model for Cleavage Plane Determination in Early Amphibian and Fish Embryos. Curr. Biol..

[50] Stewart-Savage J., Grey R.D. (1982). The temporal and spatial relationships between cortical contraction, sperm trail formation, and pronuclear migration in fertilized Xenopus eggs. Wilhelm Roux’s Arch. Dev. Biol..

[51] Murray A.W., Kirschner M.W. (1989). Cyclin synthesis drives the early embryonic cell cycle. Nature.

[52] Field C.M., Mitchison T.J. (2018). Assembly of Spindles and Asters in Xenopus Egg Extracts. Cold Spring Harb. Protoc..

[53] Bergen L.G., Kuriyama R., Borisy G.G. (1980). Polarity of microtubules nucleated by centrosomes and chromosomes of Chinese hamster ovary cells in vitro. J. Cell Biol..

[54] Petry S., Groen A.C., Ishihara K., Mitchison T.J., Vale R.D. (2013). Branching Microtubule Nucleation in Xenopus Egg Extracts Mediated by Augmin and TPX2. Cell.

[55] Ishihara K., Nguyen P.A., Groen A.C., Field C.M., Mitchison T.J. (2014). Microtubule nucleation remote from centrosomes may explain how asters span large cells. Proc. Natl. Acad. Sci. USA.

[56] Mitchison T., Kirschner M. (1984). Dynamic instability of microtubule growth. Nature.

[57] Ishihara K., Korolev K.S., Mitchison T.J. (2016). Physical basis of large microtubule aster growth. Elife.

[58] Song J.-G., King M.R., Zhang R., Kadzik R.S., Thawani A., Petry S. (2018). Mechanism of how augmin directly targets the γ-tubulin ring complex to microtubules. J. Cell Biol..

[59] Alfaro-Aco R., Thawani A., Petry S. (2017). Structural analysis of the role of TPX2 in branching microtubule nucleation. J. Cell Biol..

[60] Thawani A., Stone H.A., Shaevitz J.W., Petry S. (2019). Spatiotemporal organization of branched microtubule networks. Elife.

[61] King M.R., Petry S. (2020). Phase separation of TPX2 enhances and spatially coordinates microtubule nucleation. Nat. Commun..

[62] Alfaro-Aco R., Thawani A., Petry S. (2020). Biochemical reconstitution of branching microtubule nucleation. Elife.

[63] Bjerknes M. (1986). Physical theory of the orientation of astral mitotic spindles. Science.

[64] Dogterom M., Yurke B. (1997). Measurement of the Force-Velocity Relation for Growing Microtubules. Science.

[65] Dogterom M., Kerssemakers J.W., Romet-Lemonne G., Janson M.E. (2005). Force generation by dynamic microtubules. Curr. Opin. Cell Biol..

[66] Holy T.E., Dogterom M., Yurke B., Leibler S. (1997). Assembly and positioning of microtubule asters in microfabricated chambers. Proc. Natl. Acad. Sci. USA.

[67] Rouvière C., Houliston E., Carré D., Chang P., Sardet C. (1994). Characteristics of pronuclear migration in Beroe ovata. Cell Motil..

